# Urinary Creatinine: Barr et al. Respond

**Published:** 2005-07

**Authors:** Dana B. Barr, Samuel P. Caudill, Robert L. Jones, Christine M. Pfeiffer, James L. Pirkle, Lynn C. Wilder, Lance L. Needham

**Affiliations:** National Center for Environmental Health Centers for Disease Control and Prevention, Atlanta, Georgia, E-mail: dbarr@cdc.gov; Agency for Toxic Substances and Disease Registry, Atlanta, Georgia

Although individual predictors of urinary creatinine such as sex, body mass index, and age have been reported, no single research endeavor has documented the predictors in one study population as thoroughly as we reported in our recent article (Barr et al. 2005). The large volume of data available in the Third National Health and Nutrition Examination Survey (NHANES III; 1988–1994) [Centers for Disease Control and Prevention (CDC) 2003a] was ideal for examining and documenting these predictors. To date, our study provides the most concrete data in the literature demonstrating creatinine variation in diverse populations and the factors contributing to this variation. We agree with Gamble and Liu that although many research articles have recognized differences in creatinine concentrations within their study populations, few have attempted to correct for this variation. Our analysis of urinary creatinine concentration data in a large, representative segment of the U.S. population was intended to highlight the problems that can be encountered when routinely correcting urinary analyte concentrations for creatinine; however, Gamble and Liu point out in their letter yet another complication that may be encountered when evaluating urinary concentrations of chemicals that undergo a folate-mediated single-carbon metabolism. We are grateful that they alerted us of the possible complication of evaluating data for chemicals such as arsenic. Because folate is routinely measured in the ongoing NHANES cycles and speciated arsenic measurements have begun in the same samples, the role of one-carbon metabolism should certainly be considered in interpreting results for arsenic and other similarly metabolized chemicals for future editions of the CDC’s National Report on Human Exposure to Environmental Chemicals (CDC 2001, 2003b).
